# A Case of Drastic Reduction of Membranous Substances in the Pharynx by Interprofessional Cooperative Oral Care

**DOI:** 10.1155/2022/6375915

**Published:** 2022-03-27

**Authors:** Masahiko Okubo, Hideyuki Kamimura, Tsuyoshi Sato, Shoichiro Kokabu

**Affiliations:** ^1^Department of Oral and Maxillofacial Surgery, Faculty of Medicine, Saitama Medical University, 38 Morohongo, Moroyama-machi, Iruma-gun, Saitama, Japan 350-0495; ^2^Kamimura Dental and Orthodontics Clinic, 3-246-1, Sagami-chou, Koshigaya-shi, Saitama, Japan 343-0823; ^3^Division of Molecular Signaling and Biochemistry, Department of Health Improvement, Kyushu Dental University, 2-6-1 Manazuru, Kokurakita-ku, Kitakyusyu-shi, Japan 803-8580

## Abstract

Membranous substances in the pharynx are occasionally observed in tube feeding patients during the fiberoptic endoscopic evaluation of swallowing. Although the mechanism of the formation of these deposits sometimes causes problems, such as dysphagia, asphyxia, or aspiration pneumonia, a 91-year-old male complained about difficulty of swallowing. He had a history of cerebral infarction and aspiration pneumonitis. There was a large amount of oral desquamated epithelium, dental plaque, and calculus in his mouth. Nurses and care workers administered oral care such as rubbing the tongue and buccal mucosa daily. Dentists and oral hygienists visited and provided special oral care three times per week. At least for 77 days, the patient had no recurrence of pneumonitis. The oral desquamated epithelium and membranous substances in the pharynx decreased drastically. 2 months after the first examination, the patient was able to start rehabilitation with food. Some studies have indicated that pharyngeal deposits are derived from the oral mucosa, and through our case, we realized the importance of daily oral care by interprofessional work to reduce membranous substances in the pharynx.

## 1. Introduction

Membranous substances in the pharynx are occasionally observed in tube feeding patients during the fiberoptic endoscopic evaluation of swallowing. Although some studies have indicated that pharyngeal deposits are derived from the oral mucosa [1], the mechanism of depositing these membranous substances on the pharynx remains largely unknown. These deposits sometimes cause problems, such as dysphagia, asphyxia, or aspiration pneumonia. Membranous substances are usually removed using tweezers or aspirators. However, this method is associated with a risk of bleeding. In this paper, we report a case in which intensive oral care completely removed the pharyngeal deposits, and the patient could take food intraorally.

## 2. Case Report

**Figure 1:**
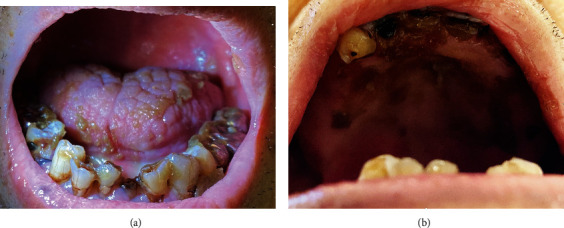
There was a large amount of oral desquamated epithelium. (a) Lower jaw and (b) upper jaw.

**Figure 2:**
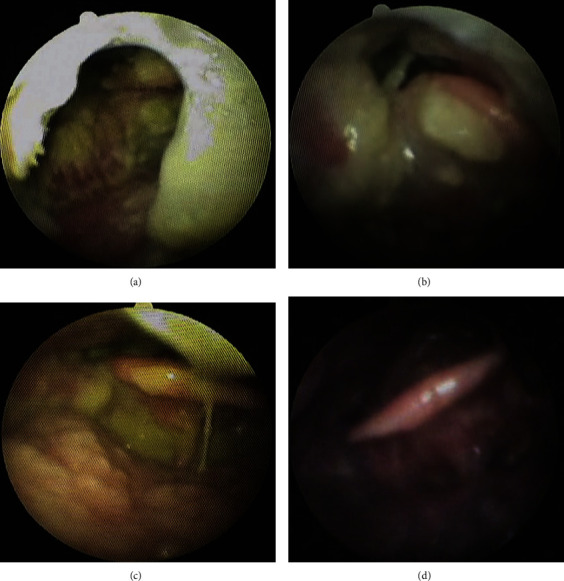
The membranous substances in the pharynx decreased gradually. Fiberoptic endoscopic pictures in the pharynx. (a) First examination, (b)14 days, (c) 37 days, and (d) 61 days after the first examination.

We visited a 91-year-old male with difficulty in swallowing. He had a history of heart failure, type 2 diabetes, bladder cancer, cerebral infarction, and aspiration pneumonitis. A month before the first visit, the patient had aspiration pneumonitis. After his pneumonitis had been treated with antibiotics, he come back to his nursing home. His physician supposed that he had severe dysphagia and then discharged with a gastrostomy feeding tube. However, his ability of swallowing was not thoroughly accessed. Therefore, his physician asked us to assess his dysphagia and provide rehabilitation for it. At the first examination, the Japan Coma Scale score was I-2. Oxygen saturation (SpO2) was 88% (reference range, ≥95%). Fiberoptic endoscopic evaluation of swallowing (FEES) revealed that there was a large amount of oral desquamated epithelium, dental plaque, and calculus in his mouth (Figure 1). We could not examine the pharynx for swallowing evaluation even during fiberoptic endoscopy due to the deposits (Figure 2(a)). The patient was deemed to require oral care, pharyngeal deposit removal, and dysphagia rehabilitation without food before initiating oral food intake. First, we changed the type of moisturizing agent from gel to liquid. Nurses and care workers administered oral care such as rubbing the tongue and buccal mucosa daily. Dentists and oral hygienists visited and provided special oral care three times per week. The patient had no recurrence of pneumonitis at least for 77 days. In addition to daily oral care, we evaluated his dysphagia by FEES on 14 days, 37 days, and 61 days after the first examination. The responsiveness, reflection, and movement of the pharynx were improved day by day. The oral desquamated epithelium and membranous substances in the pharynx also decreased drastically (Figures 2(b)–2(d)), and SpO2 improved (98%). Therefore, 2 months after the first examination, the patient was able to eat the special nonprotein foods for rehabilitation of dysphagia.

## 3. Discussion

Membranous substances in the pharynx can cause suffocation and decrease the ability to eat and swallow; hence, they are associated with aspiration pneumonia. The SpO_2_ in our patient was 88% at the first examination, but after removing the pharyngeal deposits, his SpO_2_ increased to 98%. He had no recurrence of pneumonia after the removal of the membranous substances in the pharynx.

The formation of pharyngeal deposits is closely related to membranous substances in the oral cavity. The prevalence of membranous substances in the oral cavity in tube feeding patients is 40% (10/25). Within these 40% patients, 70% (7/10) have membranous substances in the pharynx [1]. Histologically, membranous substances in both the pharynx and oral cavity are composed of cornified squamous epithelium [1–3]. Therefore, the origin of deposits in the pharynx is, at least in part, squamous epithelium, that is, membranous substances in the oral cavity, although the appearance of membranous substances in the pharynx seemed to be crust-like or sputum-like when we observed them by endoscopy [1]. Indeed, in our case, the membranous substances in the pharynx were clearly reduced in the oral cavity by intensive oral care. We speculated oral care reduced the membranous substances in the oral cavity in consequence and also reduced the substances, which had dropped into the pharynx and attached to the surrounding membrane. In addition, other mechanisms may be involved in the reduction of membranous substances in the pharynx by oral care. Because intensive oral care increases the cough reflex sensitivity in the pharynx [4] and stimulates the swallowing reflex [5] through the production of substance, of course, future research is needed to comprehend the relationship between oral care and membrane substances in the pharynx.

As described above, membranous substances in the oral cavity contain stratified squamous epithelium. Therefore, these are regarded as desquamated epithelium from the oral mucosa [1]. Dry conditions are the main risk factor for the formation of desquamated epithelium in the oral cavity [2–8]. Under dry conditions, the surface of the epithelium is denatured, and the epithelium is peeled from the lower layer [9]. Dry conditions also reduce the function of exfoliating enzymes, disturb epidermal turnover, and cause the accumulation of the stratum corneum in the oral mucosa. Therefore, the mucosa is peeled and caked under dry conditions compared to normal conditions [9, 10].

As mentioned above, since the formation of desquamated epithelium is closely related to dry mouth, the use of moisturizing agents is recommended to prevent this process in tube feeding patients [10, 11]. Oral care with moisturizing agents has been shown to prevent membranous substances at the oral membrane in tube feeding patients [3]. However, gel-type moisturizing agents should be used carefully because too much gel may contribute to the formation of membranous substances [11]. In our case, after changing the moisturizing agent from gel to liquid, the amount of membranous deposits was drastically reduced.

## 4. Conclusions

We reported that daily oral care by interprofessional workers, such as dentists, hygienists, nurses, and care workers, drastically reduces membranous substances in the pharynx and improves swallowing functions.

## Data Availability

The data that support the findings of this study are available from the corresponding author upon reasonable request.
